# Assessment of Lymph Nodes and Prostate Status Using Early Dynamic Curves with ^18^F-Choline PET/CT in Prostate Cancer

**DOI:** 10.3389/fmed.2015.00067

**Published:** 2015-09-09

**Authors:** Cédric Mathieu, Ludovic Ferrer, Thomas Carlier, Mathilde Colombié, Daniela Rusu, Françoise Kraeber-Bodéré, Loic Campion, Caroline Rousseau

**Affiliations:** ^1^Department of Nuclear Medicine, ICO Cancer Center, Saint Herblain, France; ^2^Department of Nuclear Medicine, University Hospital, Nantes, France; ^3^Centre Régional de Recherche en Cancérologie Nantes/Angers, U892, CNRS UMR 6299, INSERM, Nantes, France; ^4^Department of Medical Physics, ICO Cancer Center, Saint Herblain, France; ^5^Department of Statistics, ICO Cancer Center, Saint Herblain, France

**Keywords:** ^18^F-choline, prostate cancer, dynamic curves, early acquisition, pathological status

## Abstract

**Introduction:**

Dynamic image acquisition with ^18^F-Choline [fluorocholine (FCH)] PET/CT in prostate cancer is mostly used to overcome the bladder repletion, which could obstruct the loco-regional analysis. The aim of our study was to analyze early dynamic FCH acquisitions to define pelvic lymph node or prostate pathological status.

**Material and methods:**

Retrospective analysis was performed on 39 patients for initial staging (*n* = 18), or after initial treatment (*n* = 21). Patients underwent 10-min dynamic acquisitions centered on the pelvis, after injection of 3–4 MBq/kg of FCH. Whole-body images were acquired about 1 h after injection using a PET/CT GE Discovery LS (GE-LS) or Siemens Biograph mCT (mCT). Maximum and mean SUV according to time were measured on nodal and prostatic lesions. SUV_mean_ was corrected for partial volume effect (PVEC) with suitable recovery coefficients. The status of each lesion was based on histological results or patient follow-up (>6 months). A Mann–Whitney test and ANOVA were used to compare mean and receiver operating characteristic (ROC) curve analysis.

**Results:**

The median PSA was 8.46 ng/mL and the median Gleason score was 3 + 4. Ninety-two lesions (43 lymph nodes and 49 prostate lesions) were analyzed, including 63 malignant lesions. In early dynamic acquisitions, the maximum and mean SUV were significantly higher, respectively, on mCT and GE-LS, in malignant versus benign lesions (*p* < 0.001, *p* < 0.001). Mean SUV without PVEC, allowed better discrimination of benign from malignant lesions, in comparison with maximum and mean SUV (with PVEC), for both early and late acquisitions. For patients acquired on mCT, area under the ROC curve showed a trend to better sensitivity and specificity for early acquisitions, compared with late acquisitions (SUVmax AUC 0.92 versus 0.85, respectively).

**Conclusion:**

Assessment of lymph nodes and prostate pathological status with early dynamic imaging using PET/CT FCH allowed prostate cancer detection in situations where proof of malignancy is difficult to obtain.

## Introduction

Prostate cancer is the most common cancer in men over 50 years, and the third highest cause of death by cancer. Initial staging is reserved for tumors with intermediate or high risk according to the D’Amico classification (1). Recurrence of prostate cancer after treatment is common and occurs in 20–50% of cases at 10 years after radical prostatectomy, and in 30–40% after radiotherapy (2, 3). Management of biological relapse after curative treatment is a real diagnostic and therapeutic challenge. Hormone therapy is currently the standard palliative treatment and resistance inevitably occurs after 2–5 years (4).

In patients with intermediate or high risk, local extension, nodal, or bone metastasis, have major prognostic and therapeutic implications (4). Bone scans are recommended for patients at intermediate or high risk of progression. MRI has a major role for local extension, or location of prostate cancer after negative biopsies (5–7). Abdominal and pelvic CT can be used in order to highlight nodal metastases (8). However, imaging is supplanted by extent lymphadenectomy, which is recommended before prostatectomy or radiotherapy for intermediate and high risk prostate cancer to precisely evaluate the nodal status (9). The sentinel lymph node technique is being evaluated to guide surgeons to ensure optimal quality of dissection (10).

In prostate cancer, fluorocholine (FCH) PET/CT is indicated as an alternative to FDG PET/CT relevant to mitotic and choline kinase activities (11, 12). FCH is a highly sensitive and specific radiopharmaceutical for the initial staging of prostate cancer (intermediate or high risk tumors), or suspicion of recurrence (13–16). In most cases, PET/CT is performed in two phases (17). The first is a kinetic step centered on the pelvis to achieve the regional analysis before bladder repletion. The second consists of whole-body image acquisition 1 h post-injection.

The aim of our study was to evaluate the potential benefit of early kinetic FCH PET/CT for discriminating malignant from benign lymph node or prostate lesions, whose status has been proven by histological analysis or patient follow-up.

## Materials and Methods

### Population

Retrospective analyses were performed on patients with histologically proven prostatic adenocarcinoma and explored for initial staging or biochemical recurrence. Recurrence was defined as two consecutive PSA values of 0.2 ng/mL and above after radical prostatectomy, or three consecutive increasing PSA values above the previous PSA nadir measured at 3-month interval after radiotherapy. Patients had at least one focal FCH uptake in pelvic lymph nodes or in the prostate. All patients had an initially negative or equivocal conventional imaging, including bone scan, CT, and/or MRI. For each patient, we collected age, serum PSA, date of initial diagnosis, Gleason score, topography of prostate cancer, initial treatment, and time to recurrence. We obtained informed consent from all patients allowing the use of their clinical data for research purposes under a protocol approved in our institution.

### Acquisition and interpretation of PEC/CT

After 6 h of fasting, 3–4 MBq/kg of FCH were injected when starting PET/CT acquisition. Acquisition was realized in two phases. First a 10-min kinetic acquisition in list mode centered on the pelvis was acquired, followed by whole-body image acquisition at 60 min. Examinations were performed on two different PET/CT instruments, a GE Discovery LS (GE-LS) (GE Medical System, Waukesha WI, USA) and a Siemens Biograph mCT 40 (mCT) (Siemens, Erlanger, Germany). Images were reconstructed by iterative OSEM reconstruction with two iterations and 28 subsets associated with a 2D Gaussian filter (FWHM 5.45 mm) for GE-LS and OP-OSEM-PSF TOF with 3 iterations and 21 subsets associated with a 3D Gaussian filter (2 mm FWHM) for mCT.

Maximum and mean standard uptake values (SUV_max_ and SUV_mean_) were measured on late images in every prostatic or pelvic nodal lesion. Dynamic curves were built for each lesion, measuring the SUV_max_ and SUV_mean_ every minute in the first 10 min. To delineate the tumor contours, we used a system-specific contrast-oriented algorithm proposed by Nestle (18), using the following formula:

$$\text{SU}{\text{V}}_{\text{threshold}}=\left(k\times \text{SU}{\text{V}}_{\max}\right)+\text{SU}{\text{V}}_{\text{background}}$$

The SUV_background_ was defined as the SUV_mean_ in the gluteus maximus, and *k* was a system-specific constant determined by phantom acquisitions for each PET/CT camera. A correction of partial volume effect (PVEC) on SUV_mean_ was performed with suitable recovery coefficients (19).

### Gold standard and statistical analyses

Lymph node or prostate lesions preferentially confirmed by histology were considered (prostatectomy results, lymph node dissection, or prostate biopsies). In other cases, concordant imaging (CT or MRI) or PSA decrease after targeted radiotherapy was also used to confirm lesion status. FCH uptake in the inguinal region was interpreted as reactive inflammatory lymph node as described in the literature (20). For patients with prostatic histological proof (biopsy or resection), the agreement between PET images and histology was evaluated by comparison with the detailed histological report. Statistical analysis consisted of a comparison of repeated averages between benign and malignant lesions using an ANOVA for early acquisitions. For late acquisitions, the SUV of benign and malignant lesions were compared by comparing averages (non-parametric Mann–Whitney test). SUV between the end of the early acquisition (10 min) and the late acquisition (60 min) was compared using a paired *t*-test. Early and late acquisitions were compared using a receiver operating characteristic (ROC) curve, only for patients acquired on mCT (*n* = 71). The tests were performed bilaterally with a limit of 5% significance (*p* ≤ 0.05). The software used was SPSS 18 and Stata 13.1.

## Results

### Patients and lesions

We identified 39 patients from September 2008 to January 2014 with FCH uptake in 49 prostatic and 43 nodal lesions. Median age was 73 years and median PSA at PET time was 8.46 ng/mL (2.02–172.6). Among the 21 patients with biochemical recurrence, initial treatment was a prostatectomy for 38% (8/21) of them, and the median time to recurrence was 54.8 months (12–195). The median PSA doubling time was 8.2 months (1.7–24.7). Four patients were treated with hormone therapy at the time of PET (10.2%). Patient characteristics are presented in Table 1.

**Table 1:** **Description of patient characteristics (*n* ***=*** 39)**.

	Initial staging *n* ***=*** 18 (Min–Max)	Recurrence *n* ***=*** 21 (Min–Max)
**Median age (years)**	70.5 (51–78)	74 (55–84)
**Median PSA at PET (ng/mL)**	11.5 (3.5–172.6)	5.23 (2.02–14.95)
**Gleason score**		
6	3	5
7	9	13
8–9	6	2
Unknown	0	1
**Initial stage**		
T1	7	3
T2	6	10
T3	3	5
Unknown	2	3
**Previous treatment**		
S	NA	2
S + R		5
S + HT		1
R		10
R + HT		1
HT		2

*S, surgery; S + R, Surgery + Radiotherapy; R + HT, Radiotherapy + Hormone Therapy; HT, hormone therapy; NA, not applicable*.

Within the 39 patients explored, 19 patients (49%) had isolated prostatic lesions, 8 (20%) isolated nodal lesions, and 12 (31%) had both. Histological results were available for 96.7% (30/31) of patients with prostatic lesions and for 40% (8/20) of patients with nodal lesions (lymph node dissection). For one patient with histologically unconfirmed prostatic lesions, PSA decrease after targeted radiotherapy was considered as a proof of disease. For the 12 patients with histologically unproven lymph node lesions, malignancy was assessed by a concordant imaging between CT and MRI in five cases (25%), PSA decrease after targeted radiotherapy in three cases (15%) or inguinal lymph node location in four cases (20%). Lesion characteristics are presented in Table 2.

**Table 2:** **Description of lesion characteristics (*n* ***=*** 92) in the 39 patients**.

	Prostatic lesions number (%)		Nodal lesions number (%)	
	Per patient analysis	Per lesion analysis	Per patient analysis	Per lesion analysis
Number	31	49	20	43
Histological proof	30 (97%)	48 (98%)	8 (40%)	14 (33%)
Concordant imaging	0 (0%)	0 (0%)	5 (25%)	14 (33%)
PSA decrease after RTE	1 (3%)	1 (2%)	3 (15%)	7 (16%)

*RTE, radiotherapy*.

Within the 49 prostatic lesions, 38 were malignant (77.6%), and 28 (57.1%) were identified in patients referred for initial staging. Forty-three nodal lesions were explored, 25 were malignant (58.1%) and 18 (41.8%) were identified in patients referred for initial staging. The location of the 25 malignant lymph nodes was as follows: common iliac for 5 (20%), external iliac for 14 (56%), internal iliac for 2 (8%), obturator fossa for 1 (4%), and pre-sacral for 3 (12%). Benign lymph nodes were found in the common iliac area for one (6%), external iliac for five (28%), and inguinal node for 12 (66%).

### Early kinetic of lymph node and prostate lesions

**Figure 1:**
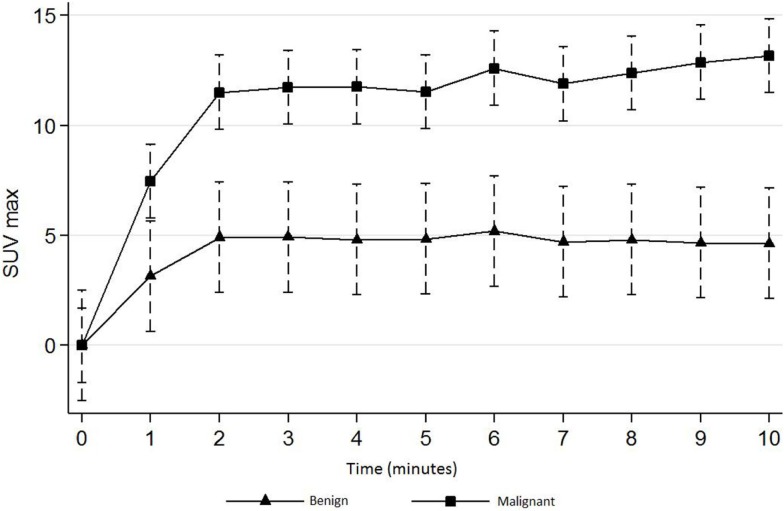
**Early dynamic curves (first 10 min) for benign and malignant lesions acquired by mCT PET/CT (*n* ***=*** 71)**.

**Figure 2:**
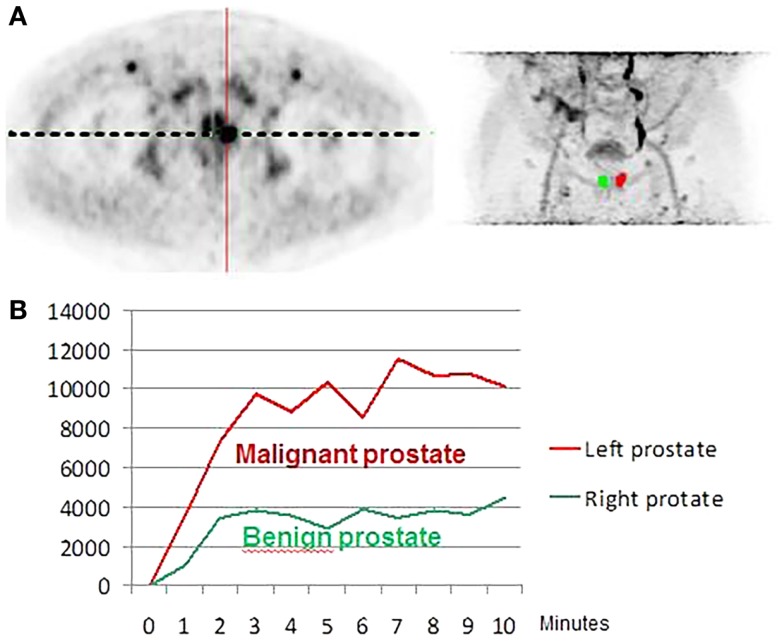
**(A)** Axial early FCH PET/CT acquisition (mCT PET/CT instrument), **(B)** maximum intensity projection (MIP) in the same patient. Patient evaluated before initial surgery with a prostatic adenocarcinoma, Gleason 3 + 4, PSA at 10 ng/mL. Histological analysis showed a left prostatic adenocarcinoma (red curve) while the right lobe was free of disease (green curve): statistically significant difference of tracer uptake between the two lobes was obtained.

The mean SUV_max_ on the early dynamic acquisitions was significantly higher for malignant versus benign lesions, respectively, on mCT (*n* = 71) and on GE-LS (*n* = 21) (*p* < 0.001 and *p* < 0.001). Malignant lesions showed intense FCH uptake, with a maximum level of SUV_max_ almost reached at the second minute post-injection, followed by a plateau. Benign lesions showed a less intense uptake. The mean SUV_max_ in the plateau was, respectively, on mCT and on GE-LS about 12 and 8 for malignant lesions and 5 and 3 for benign lesions (Figures 1 and 2). The results were confirmed with SUV_mean_ with significantly higher FCH uptake in malignant lesions (with and without PVEC).

### Analysis of 60 min post-injection images and comparison to the early kinetic results

On mCT (*n* = 71), the mean SUV_max_ in the late acquisition was significantly higher in malignant lesions versus benign lesions, 11.1 versus 3.8 (*p* < 0.001), and also on LS-GE PET (*n* = 21) 8 versus 2.7 (*p* < 0.001). There was a significant decrease of the average SUV_max_ between the end of the early acquisition and late acquisition of 13% for malignant lesions (12.8 versus 11.1, *p* < 0.001), and 19% for benign lesions (4.7 versus 3.8, *p* = 0.02).

**Figure 3:**
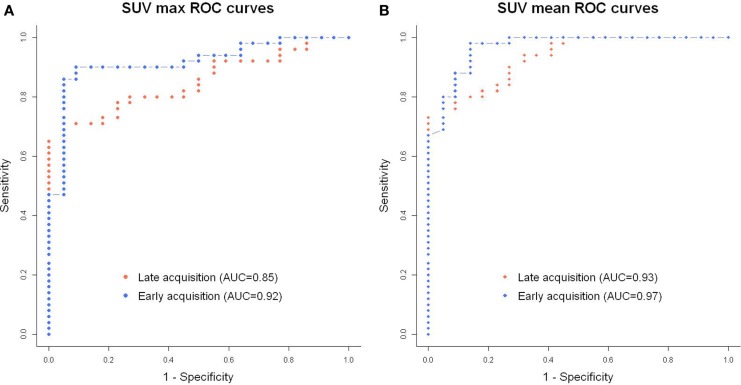
**Receiver operating characteristic curve for distinguishing benign from malignant lesions using SUV_max_ (A) and SUV_mean_ [(B), without partial volume effect correction] for patients acquired on mCT (*n* ***=*** 71)**.

On mCT (*n* = 71), the optimal SUV threshold that maximized both sensitivity and specificity in early acquisitions was obtained at 8 min post-injection. SUV_max_ ROC curve analysis showed better area under the curve (AUC) for the early acquisition at 8 min versus late acquisition (Figure 3) (0.92 versus 0.85, respectively). In the early acquisitions, SUV_mean_ displayed the best AUC in comparison with SUV_max_ and SUV_mean_ with PVEC (0.97 versus 0.92 and 0.91, respectively). The same results were found for late acquisitions with a superiority of SUV_mean_ without PVEC. Optimal threshold and data for SUV_mean_ with and without PVEC are summarized in Table 3.

**Table 3:** **Optimal threshold of SUV for distinguishing benign from malignant lesions using SUV_max_ and SUV_mean_ for patients acquired on mCT (*n* ***=*** 71)**.

	Acquisition	Optimal threshold	AUC	Sensitivity (%)	Specificity (%)	Accuracy (%)
8SUV_max_	9Early	106.1	110.92	1289.8	1390.9	1490.1
	15Late	164.3	170.85	1877.5	1977.3	2077.5
21SUV_mean_	22Early	232.5	240.97	2598	2686.4	2794.4
	28Late	292.4	300.93	3189.8	3272.7	3384.5
34SUV_mean_(PVEC)	35Early	365.1	370.91	3887.8	3981.8	4085.9
	41Late	425.0	430.87	4483.7	4572.7	4680.3

## Discussion

We conducted a retrospective study with early dynamic acquisitions of FCH PET/CT in patients explored for initial staging or suspicion of recurrence of prostate cancer. To the best of our knowledge, this is the first study to assess the kinetic uptake of the radiopharmaceutical with 1 min step images to discriminate benign from malignant lesions, and explore the potential benefit that could be provided by PVEC correction for SUV_mean_.

There are few published data on the contribution of dynamic acquisitions, and most involve small cohorts of patients. While it is known that an initial dynamic acquisition is useful in differentiating prostatic lesions from the urinary tract (21), the optimal image acquisition time remains uncertain. For prostatic lesions, Kwee et al. (17) conducted a prospective study in 26 patients with an early whole-body imaging at 7 min post-injection associated with a step on the pelvis 1 h post-injection. They showed that benign lesions could be quickly differentiated from malignant lesions with higher uptake and an increased SUV_max_ by 14% over time. Because they built a curve with two independent measurements of SUV, they could not precisely define the time to reach maximum activity. Steiner et al. (22) in a retrospective study of 15 lesions in 11 patients achieved a FCH PET/CT in 3 phases (dynamic 10 min, immediately followed by a whole body and a late acquisition on pelvis at 1 h post-injection). They found that benign lesions could be quickly differentiated from malignant lesions, which show higher uptake and an increasing SUV_max_ until the 14th minute. In our study, the dynamic acquisition in list mode allowed us to determine a maximum activity reached in about the third minute post-injection, followed by an initial plateau and a discrete reduction of SUV, especially for benign lesions.

Dynamic acquisitions with choline labeled with ^18^Fluorine are more appropriate than with ^11^Carbon, because of the short half-life of ^11^Carbon (20 min). However, a prospective study using ^11^C-Choline in 56 patients performed with two successive whole-body acquisitions at about 3–5 and 20 min post-injection, demonstrated dual phase acquisition was useful in distinguishing benign from malignant lesions (23). Nevertheless, they used a different radiopharmaceutical and they did not acquire dynamic, therefore making comparisons to our study difficult.

The behavioral differences between malignant and benign lesions, whether for prostatic or nodal lesions, encourage the use of ROC curves to define optimal thresholds to discriminate the lesions. For prostatic lesions, Kwee et al. (17) found an AUC of 0.81 at 7 min and 0.92 for late acquisition at 1 h, without defining a threshold. Oprea-Lager et al. (20) only examined nodal lesions, with an AUC of 0.93 at 30 min for the SUV_max_ and 0.92 for the SUV_mean_. They determined thresholds for lymph nodes at 2.32 and 1.66 on late acquisitions for SUV_max_ and SUV_mean_, respectively. In our study, we found similar AUC using SUV_max_ for early and late acquisitions (0.92 and 0.85, respectively). Our optimal SUV_max_ thresholds were 6.1 and 4.3 on early and late acquisitions, respectively, which allowed a sensitivity of 89.8 and 77.5%, and a specificity of 90.9 and 77.3%. We observed superior performance for SUV_mean_ than SUV_max_, for both early and late acquisitions. We also observed a better AUC for the early acquisition at 8 min versus late acquisition. Few studies explored the use of SUV_mean_, especially for response to therapy studies with a high impact of PVE correction. Nevertheless, a precise and robust delineation of tumor functional volume as used in our study, this index seems more relevant than SUV_max_. SUV_max_ provides only very limited information relating to radiotracer accumulation, and no information on the associated tumor uptake distribution or the overall tumor functional volume (24). A meta-analysis showed better repeatability performance of SUV_mean_ versus SUV_max_ and could explain the differences observed (25).

We acknowledge that our study has some limitations. First, our acquisitions used two different generation PET/CT instruments. This resulted in significant differences in detection sensitivity, making it impossible to compare patients acquired on the two different PET/CT systems. Second, as our study was based on patients where the malignant or benign status of lesions was proven, the enrollment was limited but equally important compared to other literature studies. For prostate lesions, we obtained a high rate of histological proof; 96.7% of patients had prostate lesions proven by biopsy or prostatectomy. For lymph node involvement, histological results were obtained for only 40% of patients, which was partially due to the difficulty in proposing systematic lymph node dissection based on PET/CT results. On the other hand, it was difficult to intraoperatively identify lymph node lesions detected by PET/CT because most of the time FCH positive lymph nodes did not correspond to morphologically increased node volume. Oprea-Lager et al. reported the same difficulties in obtaining proof for suspected malignant lymph nodes (20).

## Conclusion

Assessment of lymph nodes and prostate pathological status with early PET/CT FCH dynamic curves was successful in prostate cancer where proof of malignancy is difficult to obtain. In addition to avoiding bladder repletion, dynamic early acquisitions demonstrated intense and stable FCH uptake from the first minutes post-injection, and discriminated benign from malignant lesions either in prostate or in lymph nodes. SUV_mean_ without correction of PVEC and early acquisitions may perform better than maximum SUV_max_ and late acquisitions, but larger studies are warranted.

## Conflict of Interest Statement

The authors declare that the research was conducted in the absence of any commercial or financial relationships that could be construed as a potential conflict of interest.
